# The current status and evolution of hemodialysis catheters

**DOI:** 10.1080/0886022X.2025.2524523

**Published:** 2025-07-03

**Authors:** Kanan Chen, Xiaomei Liu, Qun Luo, Kedan Cai

**Affiliations:** a Department of Nephrology, Ningbo No.2 Hospital, Ningbo, PR China; b Research Center for Biomedical Materials, Ningbo Institute of Materials Technology and Engineering, Chinese Academy of Sciences, Ningbo, PR China

**Keywords:** Hemodialysis catheter, catheter design, catheter lock solution, catheter coating, catheter care, complication

## Abstract

Chronic kidney disease (CKD) represents a growing global health burden, with an increasing number of CKD patients progressing to renal failure or end-stage renal disease (ESRD). Hemodialysis is widely regarded as the usual treatment modality for patients with ESRD. Currently, more than 85% of patients initiating hemodialysis (HD) in the United States utilize a catheter for vascular access. HD is often inseparable from the use of catheters in clinical practice. Consequently, the utilization of HD catheters has risen, despite their associated complications and adverse outcomes. To minimize complications during dialysis sessions, various strategies are employed, including optimized catheter designs, specialized catheter lock solutions or functional coatings, and effective catheter care. This review outlined the current state and development of HD catheters, along with the associated adverse outcomes and effective care strategies.

## 1. Introduction

Chronic kidney disease (CKD) represents a significant global health issue. Kidney transplantation and dialysis remain the primary therapeutic options for patients with end-stage renal disease (ESRD), with kidney transplantation providing the most favorable clinical outcomes. Due to the stringent eligibility criteria for kidney transplantation, hemodialysis (HD) is generally considered the first-line treatment for patients with ESRD [1]. As of December 2023, approximately 916,000 patients in China were receiving hemodialysis treatment.

The three most common types of vascular access used in HD are arteriovenous fistula (AVF), arteriovenous graft (AVG), and central venous catheter (CVC) [2]. AVFs are associated with a lower infection rate, fewer thrombotic events, and higher long-term patency compared to other types of vascular access. However, the creation of fistula requires a highly skilled surgical procedure and a significant maturation period, typically lasting six to eight weeks, which may not be feasible for patients with a limited life expectancy [3]. Furthermore, fistulas may be prone to certain complications such as ischemic steal and congestive heart failure, when compared to catheter cannulation [4]. The AVG offers an alternative access option, particularly for patients with poor vascular conditions; however, it is associated with a higher incidence of complications than the AVF. Although CVCs are linked to a greater risk of adverse effects compared to arteriovenous access, they are still widely applied due to their convenience and patients’ preferences. In addition, catheters are frequently employed as temporary access for HD prior to fistula maturation [3,5]. In clinical practice, catheter insertion is typically performed in conjunction with fistula creation.

Historically, AVF has been the preferred option for vascular access in HD due to its lower infection rate, reduced incidence of thrombosis, and superior long-term patency [2,3]. Despite the demonstrated efficacy of permanent arteriovenous access, some dialysis patients still require permanent catheters due to challenges in establishing permanent access, limited availability of access options, and patient preference [6]. In the United States, over 85% of patients initiate HD with a catheter [7]. One study also indicated that catheters have a five-year survival rate exceeding 70%, with an overall complication rate of 30% [8], suggesting that CVC may be a preferable option for permanent dialysis access in certain specific HD patients. Furthermore, recent clinical practices emphasize a patient-centered approach to HD vascular access, considering various multiple aspects of a patient’s needs and eligibility for access, rather than strictly adhering to the ‘fistula first’ strategy [3,5,7]. The UK Kidney Association also advocates that the choice of vascular access should be guided by patients’ personal preferences, rather than the preferences of medical staff. The UK Kidney Association also recommends the use of HD catheters in very young children and patients requiring short-term HD [9].

HD catheters are classified into non-tunneled catheters for short-term vascular access and tunneled catheters for long-term vascular access. However, all types of catheters are associated with various complications, highlighting the necessity of developing new catheters to decrease complications, improve catheter patency, minimize intraluminal thrombosis, and prevent catheter kinking, collapse, or breakage [10]. This review provides an overview of recent advancements in HD catheters, with a particular focus on catheter coatings. In addition, we discuss the potential adverse outcomes that may arise during HD procession and the complication prevention role of catheter innovations.

## 2. The type of catheter

Hemodialysis central venous catheters are generally classified into two main categories: non-tunneled and tunneled. An additional type, called totally implantable dialysis catheter, has been withdrawn from the market [11]. Tunneled (long-term) catheters, composed of softer materials such as polyurethane–polycarbonate copolymer or silicone, can remain in place for weeks to months. In contrast, non-tunneled (short-term) catheters, composed of comparatively stiffer materials, require more frequent replacement, typically every few days to a week [10,12–14]. Polyurethane–polycarbonate copolymer is known for its high strength and corrosion resistance. Silicone catheters have a thicker wall to avoid kinking, but are more susceptible to erosion by povidone-iodine solutions and peroxide. As such, care should be taken to minimize catheter contact with these substances during skin sterilization [11]. In addition, non-tunneled catheters generally lack a cuff and tend to soften when exposed to body temperature. In contrast, tunneled catheters are equipped with a bonded cuff that anchors them in place, offering enhanced protection against bacterial migration [13–15].

The guidelines of the Kidney Disease Outcomes Quality Initiative (KDOQI) suggest that the use of tunneled catheters is acceptable for both short-term and long-term durations in incident and prevalent patients [5]. In contrast, non-tunneled catheters are considered appropriate only for temporary use in specific situations, such as placement for less than two weeks or in accordance with individual facility policy [5,10,16]. A retrospective, single-center study involving 413 patients who received 560 central venous catheters found that the incidence of catheter-related bloodstream infections was higher with non-tunneled jugular central venous catheters compared to tunneled jugular central venous catheters [17]. However, another study indicated that precurved non-tunneled dialysis catheters were comparable to tunneled catheters in terms of adverse events [18]. Precurved non-tunneled catheters may have the potential for long-term use, and further clinical trials are needed to validate their viability.

## 3. Catheter insertion

### 3.1. Insertion site and length

The typical sequence for HD catheter placement follows the order: internal jugular, external jugular, femoral, subclavian, and lumbar [5]. However, the catheter insertion approach may vary depending on the specific needs of each HD patient. An expert consensus recommends the ultrasound-guided supraclavicular approach of the brachiocephalic vein as the preferred cannulation method for infants and children [19]. Furthermore, certain patients may lack suitable vascular due to abnormal anatomical features or other reasons. In such case, surgical catheter implantation in the superior vena cava can provide a reliable and essential method for establishing HD vascular access, thus helping to preserve vascular access for future use [20].

The length of catheter insertion must be determined based on several factors, including the catheter insertion site, type, and patient-specific considerations [21]. The appropriate length of different HD catheters, measured from the cuff to the catheter tip, generally depends on whether the tip can reach the optimal position to ensure adequate blood flow and minimize cannulation-related complications [5,22]. For example, when puncturing the right internal jugular vein, the appropriate length is typically 19–31 cm for the tunneled catheter, with the tip positioned in the mid-right atrium. However, a length of 15 cm is sufficient to reach the cavo-atrial junction for the non-tunneled catheter. Furthermore, for femoral catheter insertion, a length of approximately 24–55 cm is required to place the tip in the inferior vena cava, ensuring sufficient blood flow [14,21,23].

### 3.2. Ultrasound-guided catheter insertion

Clinicians may also use auxiliary operations, such as real-time ultrasound guidance, to assist with dialysis catheter insertion in clinical practice. Innovations in real-time ultrasound guidance, including novel needles, the evolution of smart glasses, and 3D‑biplane probes integrated with real-time ultrasound guidance, are being actively explored [24–26]. It is recommended that real-time ultrasound guidance be utilized for HD catheter insertion, regardless of the provider’s level of experience [16]. In a prospective trial, Fournil et al. reported that ultrasound-guided internal jugular vein catheterization achieved a 96% insertion success rate with a low incidence of infection-related complications [27]. Ultrasound can also be used to assess the patency of target veins prior to catheter placement [28,29]. In addition, both ultrasound and chest radiography are necessary to confirm the tip position of the double-lumen catheter (12 F to 16 F), as well as for 8 F single-lumen catheters in some cases [23]. Nevertheless, approximately 20%–64% of catheter insertions in the internal jugular vein are still performed without ultrasound guidance, due to a lack of awareness of the use of ultrasound guidance [16,24]. Clinicians should be increasingly aware of the critical role ultrasound plays in improving the safety and success of HD catheter insertion.

## 4. The complication of hemodialysis catheter

The use of HD catheters is associated with a range of adverse outcomes, including catheter-related bloodstream infection (CRBSI), catheter-related thrombosis (CRT), catheter dysfunction, and central vein stenosis (CVS) [5,23]. These adverse effects are generally categorized into short-term and long-term complications, both of which pose significant risks to the lives of HD patients [10].

### 4.1. Short-term complications

Short-term complications have become less frequent with ultrasound guidance; however, when they do occur, they can be life-threatening for patients. These complications include arterial puncture, air embolism, central vein laceration, pneumothorax, and hemothorax [10,14]. Such mechanical complications are typically associated with the cannulation procedure and anatomical characteristics. It is important to note that the application of ultrasound guidance during insertion, combined with meticulous care throughout the procedure, has been shown to reduce the occurrence of such complications [14,30]. When these acute complications are detected, such as vascular injury, it is imperative to employ manual pressure or emergency surgery to prevent bleeding. For air embolism, it is essential to maintain the patient’s respiratory and circulatory stability, and the patient should be placed in the left lateral decubitus and Trendelenburg position to limit the movement of air bubbles [10].

### 4.2. Long-term complications

#### 4.2.1. Catheter-related bloodstream infection

For patients requiring permanent catheter placement, long-term complications are inevitable. CRBSI constitute a large proportion of long-term complications, with an incidence rate of up to 5.1 per 1000 catheter-days, leading to notable adverse outcomes in clinical settings [31]. Studies have shown that *Staphylococcus aureus* is the most common pathogen responsible for CRBSI in HD patients, with poor hygiene practices among clinicians identified as a major contributing factor to bacterial invasion [32–34]. In addition, diabetes is strongly associated with CRBSI due to the increased susceptibility and glucose-rich environment it creates [33,35]. Other factors, such as immunity deficiency and the intrinsic dangers of HD, are also recognized as key contributors to catheter-related infections [36]. Notably, the use of permanent catheters is associated with a heightened risk of metastatic infections, which is becoming increasingly prevalent. The incidence of endocarditis in HD patients is 50–60 times higher than in the general population, and despite appropriate treatment, the mortality rate remains extremely high [37]. To reduce the incidence of CRBSI and metastatic infection, using the fistula for dialysis access is highly recommended. However, a significant number of patients are not suitable candidates for fistula formation. In such cases, catheter removal combined with systemic antibiotic therapy, is an effective method for managing infection. It is generally accepted that antibiotic therapy targeting gram-positive microorganisms should be administered for two weeks. However, antibiotic resistance remains a potential issue with prolonged systemic antibiotic therapy. For patients with limited insertion sites, lock therapies without antibiotics or catheter coatings can restrict the emergence of CRBSI and antibiotic resistance, potentially avoiding the need for catheter removal [34,37–39]. Therefore, alternative preventive strategies, such as novel antibacterial lock solutions and catheter coatings devoid of antibiotics, have been developed to mitigate the risk of resistance and will be discussed in detail below.

#### 4.2.2. Catheter thrombosis

Furthermore, thrombosis, with an incidence rate of 0.8 per 1000 catheter-days, is another significant adverse outcome associated with permanent catheters. Thrombosis can result in acute catheter dysfunction, characterized by low blood flow and elevated pressure in the artery and vein lines during HD [31,40]. In clinical practice, mechanical methods (e.g., saline flush), the use of anticoagulants (e.g., heparin or warfarin), and intraluminal thrombolytic therapy with recombinant tissue plasminogen activator are commonly employed to prevent intraluminal thrombosis [10,40]. Meanwhile, as techniques evolve, catheter coatings and lock solutions with enhanced anti-thrombotic properties are increasingly playing a crucial role in preventing thrombosis.

#### 4.2.3. Poor catheter flow/catheter dysfunction

Catheter dysfunction is also a common complication in long-term HD patients, typically caused by fibrin sheath formation, thrombosis, or catheter issues [14,28]. The fibrin sheath, composed of various blood components, can block the catheter lumen to reduce the blood flow and promote bacterial colonization [36,41]. In addition to traditional treatments such as catheter removal or stripping [14], Neusser et al. demonstrated that 2% taurolidine lock solutions can effectively reduce catheter dysfunction, providing a highly cost-efficient preventive measure [42].

#### 4.2.4. Central vein stenosis

Central vein stenosis (CVS) is closely associated with subclavian vein cannulation, occurring twice as frequently as with other puncture sites, and is linked to the activation of coagulation factors because of the formation of more complicated anatomic structures [30,36,43]. CVS has the potential to impair dialysis patency, hinder AVF maturation, and contribute to superior vena cava syndrome [44]. The KDOQI guidelines recommend endovascular balloon angioplasty as the first-line treatment for patients with symptomatic CVS. However, patients with minimal symptoms or asymptomatic lesions are not considered suitable for intervention, as angioplasty may accelerate the progression of symptomatic stenosis [5]. In cases where HD central vein access is nearly exhausted, Zhao et al. also proposed a method involving percutaneous superior vena cava puncture for tunneled catheter placement. This approach demonstrated a high patency rate without the need to cannulate the left side, thereby preserving the central vein resources of HD patients as much as possible [20].

#### 4.2.5. Other catheter problems

Letachowicz et al. described a case of catheter rupture in which the efficacy of dialysis began to decline at the initiation of the procedure, despite achieving optimal flow rates [45]. Similarly, Mira et al. reported a case involving a 14-year-old single-lumen catheter, where a rupture was identified between the connector and the cuff. Additionally, a fracture at the catheter tip was observed upon its removal. Fortunately, the fractured catheter tip was promptly retrieved using an endovascular snare under fluoroscopy guidance [46]. Catheter-related issues can also lead to undetectable hemodialysis-related air embolism, which may lead to frequent cardiopulmonary collapse [47]. Although such catheter-related issues are rare in clinical practice, clinicians should remain vigilant and be prepared for this rare circumstance. Fluoroscopy plays a crucial role in detecting these infrequent adverse outcomes [46].

Overall, complications are inevitable in the use of HD catheters. Consequently, it is essential to continue research into safer and more effective treatments aimed at minimizing these complications and mitigating their potentially severe outcomes.

## 5. The catheter-related innovations

As previously mentioned, HD catheters remain a crucial component of HD treatment. Clinical staff cannot be separated from HD catheters, despite their potential limitations such as numerous complications, higher medical costs, and the restriction on complete showers compared to AVF or AVG. Therefore, it is essential to modify HD catheter to address these disadvantages and associated complications. These catheter-related innovations primarily involve improvements in catheter design (e.g., lumen, tip, and side hole design), as well as advancements in catheter surface coatings and lock solutions [48].

### 5.1. Catheter design

In 1961, Stanley Shaldon pioneered the insertion of central venous catheters into the femoral artery and vein using wire guidance. In the 1970s, Hickman introduced the formation of subcutaneous tunnel catheters. Subsequently, cuffed, tunneled silicone catheters were applied to HD patients in the late 1970s and early 1980s. Since then, catheter design has evolved rapidly, particularly from the 1990s to the present, with modifications focused on optimizing blood flow delivery, reducing the thrombosis rates, enhancing biocompatibility, increasing resistance to occlusion, strengthening resistance to antiseptic agents, and reducing the risk of catheter collapse, kinking, or breakage [49].

#### 5.1.1. Lumen

**Figure 1.**
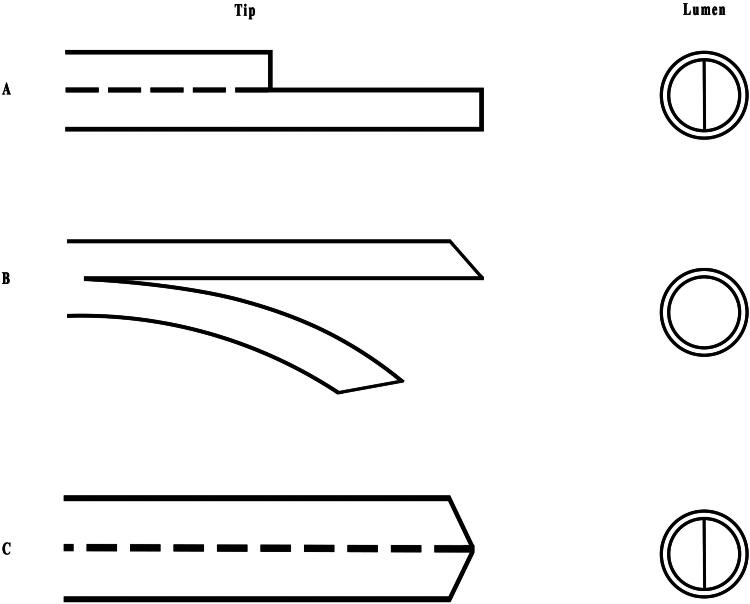
The types of catheter tips and lumens in hemodialysis catheters. (A) Step tip catheter with double-D lumen. (B) Split tip catheter. (C) Symmetric tip catheter with double-D lumen.

The design of catheter lumen has evolved from the twin single-lumen catheter to the dual-lumen catheter with a double-D design (Figure 1), which is now widely used [50]. The double-D catheter features two equally sized lumens with a reduced blood contact surface, minimizing turbulence and frictional forces against the internal wall, thereby reducing the risk of thrombosis [23]. A retrospective study by Hamid et al. found that double-lumen tunneled cuffed central venous catheters had a lower incidence of thrombus-related catheter malfunctions (22 out of 132 catheters, 16.7%) compared to the previous studies. Notably, two patients in this study had a significantly high incidence of thrombosis, with one patient experiencing complications in six out of eight catheters and the other in three out of six. This could be attributed to a thrombophilic disorder in both patients [51]. However, multi-lumen catheters may be associated with a higher risk of catheter-related bloodstream infection than single-lumen catheters, as a systematic review indicated, likely due to their relatively larger internal surface area [52]. Although Hamid et al. reported a significantly lower overall infection rate than previously reported, nearly all infected catheters in their study were removed, compared to two-thirds of catheter removals in general situations. In addition, only a few infectious double-lumen catheters in their study were effectively treated with antibiotics [51]. Therefore, greater attention should be given to dual-lumen catheters during cannulation to reduce the incidence of complications. Future trials should involve larger sample sizes and consider potential confounding factors to yield more conclusive results.

#### 5.1.2. Catheter tip

There are numerous types of HD catheters used in clinical practice, such as Quinton PermCath catheter, Mahurkar catheter, Ash Splitcath catheter, Tal Palindrom catheter and so on [53]. The tips of these catheters are mainly classified into three types: step tip, split tip, and symmetric tip (Figure 1) [50]. In addition to the commonly used types, the Canaud-Tesio catheter features two separate single-lumen catheters with surrounding side-holes, which help maintain blood flow after attachment to the blood vessel wall. However, this type of catheter requires individual placement of each catheter, which makes it less favored by many clinicians [54]. Moreover, the CentrosFlo self-centering catheter is a modified version of the split-tip catheter. It may prevent fibrin sheath formation and ensure optimal blood flow due to its inwardly directed port. With limited data available, further clinical trials are needed to support its broader application [10,53].

Recirculation generally occurs when the input lumen extracts dialyzed blood directly from the outflow lumen, thereby reducing hemodialysis efficiency. The application of the split-tip catheter, which features two separated distal tips, has been shown to reduce the occurrence of recirculation [23]. In addition, catheters with symmetric tips and biased ports may result in less recirculation than other designs [55]. Two other studies also demonstrated that symmetric tips perform better in preventing recirculation when blood flow is reversed [56,57]. However, Ling et al. noted that different catheter tips offer distinct advantages in the clinical setting, with similar risks of catheter-related complications such as infection and thrombosis. Furthermore, catheters with symmetric tips tend to be more expensive than those with step or split tips [58]. Therefore, the optimal catheter tip design remains undetermined at present.

#### 5.1.3. Side hole

Side holes at the end of the catheter have been found to improve HD blood flow and reduce recirculation; however, they are also associated with an increased risk of clotting and infection. David et al. found the symmetric-tip catheters with medium-sized, oval-shaped, and parallel side holes perform best in terms of blood flow shear stress, which is the key factor in platelet activation [59]. Furthermore, side holes can operate effectively even when the distal aperture is blocked by thrombus or adheres to the vascular wall. For instance, the split tip catheter features side holes on each limb to ensure that some holes remain away from the vessel wall, preventing complete access blockage. As previously mentioned, while side holes help facilitate catheter patency, they also increase the risk of clotting and infection due to the formation of relative dead space at the tip [55]. Michael et al. found that catheters placed in goats’ jugular veins without side-holes exhibited a lower risk of clotting. Furthermore, clots in the non-side-hole catheters could be aspirated more completely, maintaining durable patency without the need for catheter removal [60]. Another study, which followed 60 patients with symmetric-tip non-side-hole catheters, observed that the incidence rate of catheter-related bloodstream infection (CRBSI) was lower than the target rate set by the National Kidney Foundation Kidney Disease Outcomes Quality Initiative guidelines [61]. Consequently, side holes represent a double-edged sword. A comprehensive evaluation of the benefits and potential adverse outcomes of side holes in the HD catheter is crucial for clinicians to ensure optimal patient care.

#### 5.1.4. Novel catheter design

**Figure 2.**
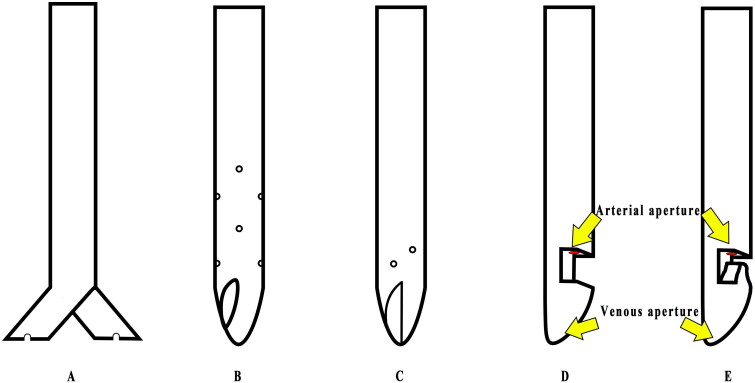
The novel design of HD permanent and temporary catheter. (A) Pristine HD catheter. (B) Glidepath^TM^ HD catheter. (C) Vectorflow catheter. (D) Uplift shape tip temporary catheter. (E) Branch shape tip temporary catheter.

Numerous innovative catheter designs have been reported to offer enhanced benefits for HD patients (Figure 2). For example, the Pristine HD catheter features a split, symmetrical tip design without side holes. This free-side-hole design reduces clot formation compared to traditional symmetric tip catheters [62]. The Glidepath^TM^ HD catheter has a symmetrical tip with 360° side holes and curved distal apertures on opposing sides, designed to prevent catheter occlusion. And the Vectorflow catheter boasts a symmetrical tip with helically arranged apertures, promoting spiral laminar blood flow that helps prevent recirculation [63]. In addition, short-term HD catheters with uplift or branch tips have been demonstrated to enhance overall efficiency while reducing the incidence of recirculation. [64]. Although various catheter designs are available on the market, clinical evidence regarding optimal catheter design remains limited. Therefore, the choice of HD catheter should be based on clinical judgment and experience.

### 5.2. Catheter coating

It is widely acknowledged that HD catheters are associated with higher infection rates and more frequent thrombosis events compared to other forms of HD vascular access; however, they are still commonly used in the current clinical setting. Several measures have been implemented to mitigate these complications, including the use of catheter coatings, the impregnation of catheters with antiseptics or antibiotics, and the development of catheter designs [65]. Moreover, the material of HD catheters tends to adsorb proteins, which facilitates the development of thrombosis. This process also plays a crucial role in biofilm formation, infections, and the eventual onset of bacteremia [66]. Therefore, there is an urgent need for catheter coatings that offer improved biocompatibility and multifunctionality in current clinical practice [13,67]. At present, heparin, antibiotics and catheter removal are used to prevent aforementioned adverse outcomes. However, these interventions may result in complications such as bleeding, antibiotic resistance, and high medical expenditure [38,39,41,68]. To minimize the occurrence of these side effects, ongoing research and development are focusing on novel catheter structures and coatings.

Catheter coatings commonly used in clinical practice can be classified into three categories: leaching coating, non-leaching coating, and nonfouling coating. The duration of effectiveness is a critical property of catheter coatings (Table 1). The longevity of leaching coatings depends on active substance released. Antibiotic coatings are typically applied to temporary catheters that are in place for more than five days [11,13,69], given the potential for severe side effects. Metal-release coatings offer excellent antimicrobial properties due to continuous metal ion release for at least 16 days without safety risks [70]. It has been reported that heparin coatings can preserve catheter patency for at least one month [71]. Non-leaching or nonfouling coatings generally rely on physical mechanisms to perform their function, resulting in a duration of effectiveness that typically exceeds two weeks. [72,73].

**Table 1.** The advantages, risks and duration of effectiveness of different catheter coating types.

The types of catheter coating		Advantages	Risks	Longevity
Leaching coating	Antibiotic	Infection prevention	Antibiotic resistant	Less than two weeks
	Metal	Infection and clot prevention	Metal toxicity	More than two weeks
	Heparin	Clot prevention	Bleeding	More than one month
Non-leaching coating		No drug resistant	No serious side effects	More than two weeks
Nonfouling coating		Bacterial adhesion reduction Clot prevention	Infection under long-term placement	More than one month

#### 5.2.1. Leaching catheter coating

**Figure 3.**
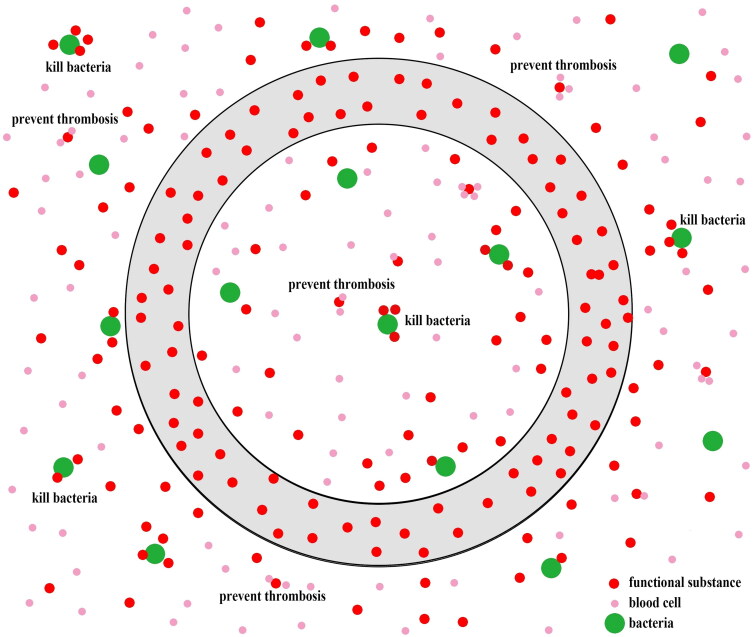
Leaching catheter coating releases functional substances around the catheter such as antibiotics or heparin to kill the bacteria and avoid thrombosis.

Leaching catheter coatings (Figure 3) have the potential to release functional substances into the bloodstream to kill bacteria or prevent thrombosis. However, they may also contribute to allergic reactions or the development of antibiotic resistance [74]. Common substances used in catheter coatings include antibiotics, heparin, and metals [13], particularly in non-tunneled catheter insertions. Catheters impregnated with chlorhexidine and silver sulfadiazine or with minocycline and rifampicin, are frequently used to inhibit microorganism adhesion and biofilm formation. Nevertheless, antibiotic resistance remains a significant concern with antibiotic-based coatings [11,67,74,75]. Therefore, the antibacterial coatings without antibiotics present a promising solution to this issue. Silver coating is the most commonly used metal coating for HD catheters [76], despite the risks of toxicity associated with the release of excessive metal ions. In contrast, copper is an essential element in the human body, which suggests that copper-coated catheters may present a lower risk of toxicity [67]. The inherent benefits of copper ions have led researchers to explore novel coating approaches incorporating copper. The study has shown that the copper-grafted chitosan coatings exhibit excellent capabilities in preventing bacterial colonization and mitigating inflammatory reactions after bacterial implantation, both *in vitro* and *in vivo* [77]. Besides its antibacterial properties, Wang et al. also reported that fewer platelets adhere to the copper-bearing coating, which may be attributed to the ability of the copper ions to disrupt platelet structure [67]. Moreover, some studies have incorporated herbal extracts, such as *Cinnamomum*, *Achillea millefolium L.* (yarrow), into catheter coatings to enhance their antibacterial properties. These extracts have the potential to serve as novel antibacterial agents for leaching catheter coatings [78,79].

#### 5.2.2. Non-leaching catheter coating

**Figure 4.**
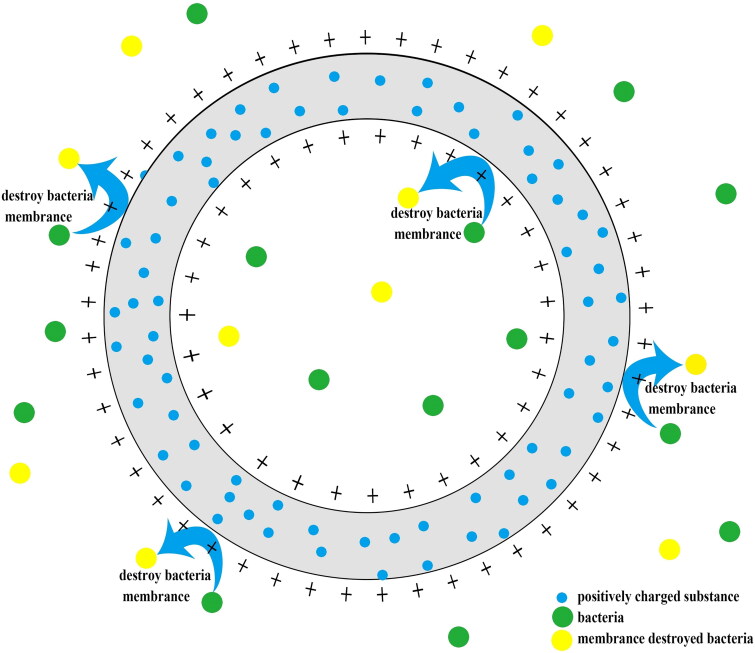
Non-leaching catheter coating relies on coating filled with positive charges surface to destroy the membrane of bacteria and resist infection.

Non-leaching catheter coatings (Figure 4) exert antimicrobial effects through their positively charged surfaces, which disrupt bacterial cell membranes. Interestingly, no adverse events have been reported to date with this coating [74,80]. Gao et al. conducted a study involving mixed-charge polypeptide deposited on catheters, which demonstrated exceptional antifouling properties along with excellent antibacterial and antibiofilm activities by rupturing bacterial cell membranes [81]. Similarly, Krikava et al. reported that non-leaching coating significantly reduce the incidence of bloodstream infection [80]. Since these type of catheter coatings rely on physical mechanisms to exert antimicrobial and antithrombotic effects, drug resistance is less likely to occur compared to antibiotic-leaching coating [81]. These findings highlight a promising trend in the development of non-leaching catheters for clinical applications.

#### 5.2.3. Nonfouling catheter coating

**Figure 5.**
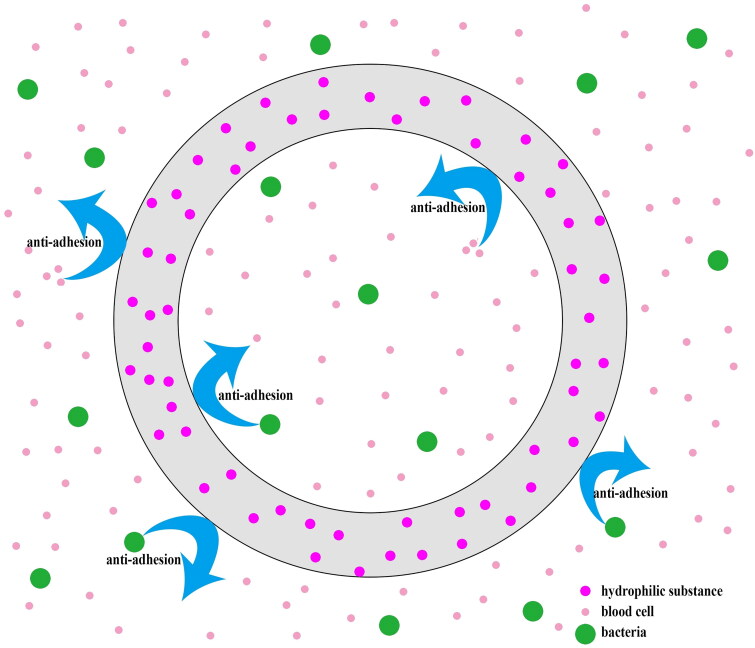
Nonfouling catheter coating prevents bacteria or blood cell adhesion *via* a smoothly hydrophilic surface.

Hydrophilic catheter coatings, also known as nonfouling coatings (Figure 5), have emerged as a promising strategy in modern catheter technology. The smooth, hydrophilic surface of these coatings prevents bacterial adherence and thrombosis [82]. However, due to their inability to eradicate bacteria, the accumulation of bacteria within the catheter may increase over time [83,84]. Liquid perfluorocarbon coating, regarded as a novel antibiotic-free fluoropolymer catheter coating, are used to prevent thrombosis, bacterial attachment, and colonization because of the slippery surface of the catheter wall. However, the complex coating process and high costs may limit the widespread clinical use of liquid perfluorocarbon coating [75]. In addition, the deaths of HD patients due to perfluorocarbon residues in 2001 highlight the need for clinicians to exercise caution when using this coating [85,86]. Polymerized coatings incorporating hydrophilic zwitterionic molecules have demonstrated excellent consequences in preventing catheter complications and reducing mechanical damage during catheter insertion, owing to their minimal friction [73]. Another effective anti-adhesion method, known as water-infused surface protection, has been proposed to reduce fibrin sheath formation by creating a boundary layer of flowing water between the blood flow and the catheter wall [66]. To further combat bacterial contamination that may arise with long-term exposure, sterilization processes could be combined with nonfouling catheter coatings to reduce the potential for bacterial colonization [81,84]. The development of novel coating biomaterials offers more options to prevent catheter-related complications in HD patients.

#### 5.2.4. Challenges of catheter coating

Although the utilization of catheter coatings offers many benefits to HD, it is not always the optimal solution. A large-scale prospective study found no significant difference in the prevention of infection and thrombosis between peripherally inserted central catheters with antimicrobial and antithrombogenic coatings and standard catheters. This result contrasts with findings from other studies, likely due to variations in post-insertion and the excessively rapid clinical adoption of these ‘new’ technologies [87]. Moreover, another study indicated that antimicrobial catheter coatings may not reduce the incidence of catheter-related bacteremia in the HD population, given the different situation of HD [88]. However, this does not imply that innovation in catheter coatings is futile. Further clinical research is crucial to assess the clinical reliability of these novel coatings. Moreover, clinicians must adhere to rigorous operating standards when utilizing these novel catheters.

Despite advancements in catheter coatings and the emergence of new technologies offering more clinical options for HD, challenges such as antibiotic resistance, cytotoxicity [67], allergic reactions [11,74], and cost-efficiency [75] remain significant obstacles to the widespread use of HD catheter coatings. Further research is essential to enhance the economic feasibility and practical effectiveness of dialysis catheters. Concurrently, it is crucial to improve the clinical evaluation before adopting novel catheter coatings and to place greater emphasis on basic HD care when utilizing these innovations.

### 5.3. Catheter lock solution

Hemodialysis catheters carry a high risk of CRBSI in clinical practice. The primary treatments of CRBSI involve removing the infected catheters and administering high doses of systematic antibiotics [38,39]. However, these strategies can impose a significant economic burden on both hemodialysis patients and the medical system [41]. To address this challenge, medical workers utilize catheter lock solutions as an alternative to catheter removal, helping to reduce medical expenses. Meanwhile, catheter lock solutions are employed to prevent thrombosis after each dialysis session. It is well-established that the lock solutions offer significant benefits to patients undergoing long-term HD treatments. We will describe the different types of lock solutions below and summarize their advantages and disadvantages in Table 2.

**Table 2.** The advantages and risks of different catheter lock solutions.

Types of lock solution	Advantages	Risks
Heparin	Widely application Thrombosis reduction	Lack antibacterial property Bleeding
Antibiotic	Widely application Infection reduction	Presence of toxicity and drug resistance
Alcohol	Cheap Antibacterial property on biofilm-producing bacterial or fungi	Concentration-dependent thrombosis
Citrate	Bleeding prevention Clot and infection prevention	Hypocalcemia
Sodium bicarbonate	Cheap Clot and infection prevention	Hypocalcemia
Taurolidine	Clot and infection prevention	Potential dose-dependent hepatotoxicity

#### 5.3.1. Heparin combined with antibiotics lock solution

Currently, 1000 IU/ml heparin solution is frequently applied in clinical practice to prevent thrombosis. However, the heparin lock solution may increase the risk of bleeding and heparin-induced thrombocytopenia, which can be harmful to hemodialysis patients [68]. Moreover, since heparin does not have antimicrobial properties, heparin lock solutions are often combined with antibiotics to enhance their antibacterial effectiveness. A study has indicated that antibiotics combined with low-dose heparin (500–2500 U/mL) may have a better preventive effect than heparin alone for CRBSI and bleeding (RR, 0.20 vs. 0.94 and 0.17 vs. 0.38, respectively) [89]. In addition, Zhang et al. proposed that the combination of gentamicin and heparin is the optimal choice for preventing CRBSI. However, gentamicin is known to be associated with ototoxicity [90]. Hereby, long-term prospective studies are needed to investigate the potential adverse outcomes of this combination.

#### 5.3.2. Alcohol lock solution

Although antibiotic lock solutions are effective in controlling bacterial infections, they are unable to eliminate certain bacteria or fungi prone to biofilm formation. In this context, 70% alcohol lock solution has demonstrated excellent sterilization properties for biofilm-forming microorganisms [91]. The combination of 70% alcohol with anticoagulants (such as heparin and 4% citrate) has been shown to reduce the rate of CRBSI by 57%, while also being highly cost-effective [68]. However, excessive concentrations of alcohol lock solution may lead to the deposition of plasma proteins, thereby increasing the risk of thrombus formation. Further studies are needed to determine the optimal alcohol concentration and dwell time, or to explore the use of alcohol in combination with other anticoagulants to address this issue [68,92].

#### 5.3.3. Citrate lock solution

Sodium citrate has been identified as a calcium ions binding substance that inhibits thrombosis and possesses some sterilizing properties. 4% trisodium citrate lock solution can effectively reduce the risks of bleeding and infection that appear in heparin frequently in long-term HD catheters [93]. However, there was no significant difference in the incidence rate of CRBSI between high (46.7%) and low (4%) concentrations of citrate lock solution (HR 1.10, 95% CI 0.74–1.64, *p* = .64) [94]. Furthermore, high concentrations of citrate solution lead to localized hypocalcemia, potentially damaging the tissues and organs around the catheter tip. Even the death of an ESRD patient after applying a 47% citrate lock solution may be closely related to citrate-induced hypocalcemia [68]. Therefore, a 4% citrate lock solution is clinically recommended over higher concentrations like 46.7%.

#### 5.3.4. Sodium bicarbonate lock solution

Researchers have also focused on inexpensive sodium bicarbonate, which has been demonstrated to inhibit biofilm formation and surface adhesion. A trial demonstrated that normal saline lock solution had 26.6 and 15.9 times higher odds of catheter loss due to thrombosis and infection, respectively, compared to sodium bicarbonate lock solution [41]. Another study showed that the safety of non-tunneled catheter with sodium bicarbonate is similar to those treated with antibiotics [95]. Furthermore, since bicarbonate exerts its anticoagulant effect by binding to calcium ions [41], bicarbonate lock solutions have the potential to induce hypocalcemia. More clinical evidence is required to further support its application.

#### 5.3.5. Taurolidine lock solution

Taurolidine disrupt the integrity of bacterial cell membranes and resist thrombosis by decreasing the activity of coagulation factors. In addition, taurolidine lock solution has been shown to result in a lower incidence rate of catheter removal in tunneled or non-tunneled catheters compared to citrate lock solution (HR 0.34, 95% CI 0.19–0.64, *p* < .001) [94]. Agarwal et al. also found that taurolidine (13.5 mg/ml) combined with heparin (1000 units/ml) reduced the risk of CRBSI by 71% versus standard heparin (1000 units/ml) [96]. It has been reported that this lock solution does not cause severe side effects. Nevertheless, certain experimental studies claimed that taurolidine may be associated with dose-dependent direct toxic effects on hepatocytes [97]. Consequently, further clinical research is needed to assess the potential for liver injury associated with taurolidine lock solutions.

#### 5.3.6. Other new lock solution

With advancements in technology, novel theories and materials are increasingly being applied in the catheter lock solutions. The nitric oxide donor molecule S-nitrosoglutathion has shown exceptional antimicrobial activity and thrombosis prevention. Morgan et al. found that the S-nitrosoglutathion lock solution could inhibit biofilm formation and clot development without causing hemolytic or cytotoxic effects [98]. It is important to note that these novel materials have been scarcely explored as lock solutions for HD catheters. For example, S-Nitroso-N-acetyl-L-cysteine Ethyl Ester (SNACET) has been extensively studied as an oral therapeutic, yet its use in catheter lock solutions remains unexplored. When incorporated into the lock solution, SNACET has demonstrated broad-spectrum antibacterial properties for at least 18 h [38]. In a word, advancements in lock solutions are continuously driven by progress in medical technology and theoretical developments.

## 6. Catheter care

In the context of the widespread hemodialysis application, comprehensive care is a critical process that complements catheter designs, materials, and techniques to improve vascular access outcomes [12]. In clinical practice, appropriate care measures can significantly reduce the morbidity and the risks associated with infections [99]. For example, a dedicated multidisciplinary care team can address the diverse needs of HD patients while also reducing medical costs [100,101]. Moreover, repeated education of patients and medical staff on aseptic techniques and proper vascular access care plays a crucial role in improving clinical setting [5,102,103]. The innovative use of simulation-based mastery learning has the potential to increase clinicians’ success rate in catheter insertion procedures and is expected to enhance the levels of HD education in the future [104,105]. More importantly, it is essential to recognize the pivotal role of dressings and ointments at the catheter exit site. Chlorhexidine gluconate dressings have been shown to reduce the incidence of infection, and mupirocin or povidone iodine ointments are recommended for use at the catheter exit site [5,106,107]. Even the involvement of medical leadership can prevent the reverse outcomes during HD sessions [102,108]. Effective catheter care not only reduces the incidence of complications but also enhances the overall quality of HD treatment.

## 7. Summary

With the increasing understanding of hemodialysis, the recommendation for vascular access has shifted from the ‘fistula first’ to the ‘patient preference first’ or ‘right access’. In this context, it is an irrefutable fact that catheters play a more and more vital role in the treatment of HD. However, the frequent occurrence of catheter complications obstructs the long-term use of traditional catheters in HD patients. Therefore, there is an urgent need to explore the new forms of HD catheters.

Currently, various innovative catheter designs are proposed, many of which exhibit excellent antithrombotic properties and effectively prevent recirculation. Because each catheter design possesses its own unique advantages, the selection of the appropriate catheter needs to be based on the specific clinical settings and the clinician’s experience. In addition, HD catheters with novel material coatings have also been highly successful in preventing CRBSI and thrombosis. It is important to note that as innovative HD catheters continue to be developed, their safety and reliability must be thoroughly evaluated. Moreover, even certain mature research in other fields may yield unexpected effects when applied to HD catheters.
